# Temporal Expression Patterns of Clock Genes and Aquaporin 5/Anoctamin 1 in Rat Submandibular Gland Cells

**DOI:** 10.3389/fphys.2017.00320

**Published:** 2017-05-23

**Authors:** Ryouichi Satou, Masaki Sato, Maki Kimura, Yoichi Ishizuka, Masakazu Tazaki, Naoki Sugihara, Yoshiyuki Shibukawa

**Affiliations:** ^1^Department of Epidemiology and Public Health, Tokyo Dental CollegeTokyo, Japan; ^2^Department of Physiology, Tokyo Dental CollegeTokyo, Japan

**Keywords:** aquaporin, circadian rhythm, clock gene, secretion, chloride channel, transport

## Abstract

Circadian rhythms are essential for health and regulate various physiological functions. These rhythms are regulated by a negative-feedback loop involving clock genes in the suprachiasmatic nucleus (SCN) and peripheral tissues. The rate of secretion of salivary substances, ions, and water follows a circadian rhythm, however, the relationship between the molecular mechanism of salivary secretion and peripheral circadian rhythm is not yet clear. Anoctamin 1 (ANO1, also known as TMEM16A) and Aquaporin 5 (AQP5) play an important role in the transport of ions and water in the submandibular glands (SGs). We examined the interaction between the rhythmic expression pattern of the clock genes, *Ano1* and *Aqp5*, in rat whole SGs as well as isolated acinar and ductal cells. Circadian rhythmic expression for *Bmal1, Per1, Per2, Clock, Cry1, Cry2, Ror*α, and *Rev-erb*α mRNAs, also called the clock genes, was observed in rat SGs by semi-quantitative RT-PCR analysis. We also observed rhythmic patterns in *Ano1* and *Aqp5* mRNA expression. The expression of ANO1 protein also showed circadian rhythm, as confirmed by western blot analysis. We could not observe any time delay between the peak expression of ANO1 protein and its mRNA. Expression levels of the clock gene mRNAs in the ductal cells was higher than that in acinar cells, however, rhythmic oscillations were observed in both. Our results suggest that SGs have peripheral clocks, and rhythmic expressions of *Ano1* and *Aqp5* along with the clock genes, may play an important role in the circadian regulation of salivary secretion.

## Introduction

Circadian rhythm is essential for driving various physiological and metabolic functions in most living organisms, and coordination of their behavioral and biological processes such as sleep/wake cycles, body temperature, hormone secretion, blood pressure, and salivary secretion (Reppert and Weaver, 2002). Disturbance of the circadian rhythms can cause major systemic conditions such as cancers, central nervous system diseases, cardiovascular disorders, metabolic syndromes, and mental illness (Hastings et al., 2007). The rhythm is regulated by a master clock and other peripheral biological clocks. The master clock generates 24-h circadian rhythms in all mammals. It is located in the suprachiasmatic nucleus (SCN) of the hypothalamus and orchestrates the regulation of peripheral clocks. Several other peripheral clocks are regulated independently by endogenous self-oscillating transcription factors called the clock genes. Molecular regulation by the clock genes in the circadian oscillator is based on interconnected transcriptional/translational feedback loops (Reppert and Weaver, 2002; Suárez-Trujillo and Casey, 2016). Among them, aryl hydrocarbon receptor nuclear translocator-like protein 1 (*Arntl*/*Bmal1*), period (*Per*), circadian locomotor output cycles kaput (*Clock*), and cryptochrome circadian clock (*Cry*) are termed as the core clock genes. Retinoic acid receptor-related orphan receptor alpha (*Ror*α) and nuclear receptor subfamily 1 group D member 1 (*Rev-erb*α) regulate the transcription of *Bmal1*. The key transcription factors, CLOCK and BMAL1, form heterodimers that bind to the enhancer box (E-box) sequences and activate transcription of the *Per* and *Cry* genes. The PER and CRY proteins subsequently repress the transcription at their own promoters through a negative feedback loop, effected by acting on the CLOCK–BMAL1 complex (Shearman et al., 2000; Sato et al., 2014). This feedback loop controls the master and the peripheral clocks in most tissues.

Salivary flow rate or the secretion rate of salivary substances such as Na^+^, ${\text{HCO}}_{3}^{-}$, Cl^−^, K^+^, and α-amylase also follows a circadian rhythm (Dawes, 1972). Especially, it is known that unstimulated salivary flow rate is extremely low during sleep (Dawes, 1972). Recent studies have shown the circadian expression rhythm of clock gene (*Per1, Per2, Bmal1, Cry1*, deleted in esophageal cancer 1[*Dec1*], *Dec2*, D-site of albumin promoter binding protein [*Dbp*] and *Rev-erb*α) and *amylase 1* in SGs (Furukawa et al., 2005). The localization of core clock proteins (*Bmal1, Per2*, and *Clock*) and *Bmal1* and *Per2* mRNAs in the mucous acini and striated ducts of salivary glands was also determined by *in situ* hybridization (Zheng et al., 2012). Moreover, light and food entrainment control the phase of submaxillary *Per1* expression (Vujović et al., 2008). These studies suggest that not only acinar but also ductal cells play an important role in circadian oscillation of salivary secretion. Although the findings imply that clock genes influence the physiological functions in the salivary glands, detailed rhythmic and temporal expression patterns of the clock genes in salivary gland cells, that is, acinar and ductal cells, remain to be investigated. The major salivary glands, SGs as well as the parotid and sublingual glands normally contribute over 90% to the total volume of unstimulated saliva. Percentage contributions of salivary glands during unstimulated saliva are as follows: 65% from SGs, 20% from parotid, and <10% from sublingual and minor glands (Humphrey and Williamson, 2001). SGs are mainly composed of two epithelial cell types: the acinar cells, which secrete water, ions, and the salivary proteins; and the ductal cells, which modulate the ionic composition of the saliva (Humphrey and Williamson, 2001).

There are transcellular and paracellular transport pathways for the secretion of water and ions in the SGs, which are driven by changes in transmembrane osmosis and water channel gating (Turner and Sugiya, 2002). Recent studies have shown that Anoctamin 1 (ANO1) and Aquaporin 5 (AQP5) play an important role in water and ion transport in SGs (Ma et al., 1999; Yang et al., 2008). ANO1 and AQP5 are localized on the apical membrane of the SGs (Yang et al., 2008). ANO1, is a transmembrane protein that functions as a Ca^2+^-activated chloride channel (CaCC). CaCCs control the apical Cl^−^ efflux, which is essential for the vectorial transport of electrolytes and water in the bronchiolar epithelial cells, pancreatic acinar cells, proximal kidney tubule epithelium, retina, dorsal root ganglion sensory neurons, airways, and salivary glands (Caputo et al., 2008; Yang et al., 2008; Ferrera et al., 2010). AQPs are channel proteins that regulate the transmembrane movement of water in response to osmotic gradients for driving the salivary secretions. In SGs, AQP5 is one of the major aquaporins expressed on the apical membrane of the acinar and intercalated ductal cells (Delporte and Steinfeld, 2006; Matsuzaki et al., 2012). Although *Ano1* and *Aqp5* are key genes required for water and ion secretion in the SGs, their exact contribution to the regulation of circadian rhythm in salivary secretion remains to be validated. In the present study, we examined the interaction of temporal rhythmic expression patterns among the clock genes *Ano1* and *Aqp5*, in rat SG acinar and ductal cells.

## Materials and methods

### Ethical approval

All animals were treated in accordance with the Guiding Principles for the Care and Use of Animals in the Field of Physiological Sciences, approved by the Council of the Physiological Society of Japan and the American Physiological Society. All animal experiments were carried out in accordance with the Guidelines for the Treatment of Experimental Animals at Tokyo Dental College. All the experimental protocols were approved by the Ethics Committee of Tokyo Dental College (No. 280901).

### Animals

Six to eight weeks old, male Wistar rats (Charles River Laboratories Japan, Inc., Tsukuba, Japan) were used. Only male rats were chosen, to avoid the effect of sex-related hormonal differences on the circadian rhythm. Animals were housed with 12:12-h light/dark cycle (lights on from 08:00 to −20:00) with food and water available *ad libitum*. Before all experiments, we confirmed that rats are acclimated to these light/dark conditions by monitoring the daily pattern of wheel-running activity for 14 days (data not shown). All experiments were performed in accordance with Zeitgeber time (ZT, referring to an objective time scale wherein ZT0 is set as the time of lights on and, ZT12 is set as the time of lights off.), with ZT0 set as 08:00.

### Isolation of rat SG acinar and ductal cells

Submandibular acinar and ductal cells were isolated as previously described (Nezu et al., 2000; Sakai et al., 2002) with modification. Detailed methods are described in the Appendix. SGs were dissociated from anesthetized rats at each time point (ZT0 to ZT48). All cell isolation protocols were carried out within 2 h, and cells obtained (acinar and ductal populations) were immediately subjected to mRNA expression analysis or measurement of kallikrein activity.

### Measurement of kallikrein activity

Kallikrein activity was measured (Supplementary Figure 1 and Supplementary Table 3) according to the method by Geiger et al. (1980), using *N*α-benzoyl-dl-arginine *p*-nitroanilide. Detailed methods are described in the Appendix.

### Relative mRNA abundance analysis using real-time semi-quantitative RT-PCR (sqPCR)

Both sides of SGs were dissociated from anesthetized rats at ZT0, ZT6, ZT12, ZT18, ZT24, ZT30, ZT36, ZT42, and ZT48, and total RNA was immediately extracted by a modified acid guanidium phenol-chloroform method following SG dissociation. For cell isolation, both sides of SGs were dissociated at ZT0 and at 6-h intervals from ZT0 to ZT24. Isolated acinar and duct-like cells were then subjected to total RNA extraction within 2 h after whole SG dissection. For these samples, sqPCR analysis (Thermal Cycler Dice, TaKaRa Bio) was performed. For all series of sqPCR analyses, the same quantity of total RNA (50 ng) was used. Expression level of the internal reference gene, β*-actin*, was measured using One Step SYBR® PrimeScript® RT-PCR Kit II (Perfect Real Time, TaKaRa Bio), using probes labeled with 6-carboxyfluorescein (6-FAM). The primers used are described in Supplementary Tables 1, 2. sqPCR analysis was performed using the comparative Ct method (2^−ΔΔCt^, where Ct implies cycle threshold). This method was used to assess relative expression level of mRNA normalized to β*-actin*, using the Thermal Cycler Dice real time system software version 5.11.

### Western blot analysis

Both sides of SGs were dissected at ZT0, ZT6, ZT12, ZT18, ZT24, ZT30, ZT36, ZT42, and ZT48, and immediately stored in liquid nitrogen. The tissue was homogenized in ice cold radioimmunoprecipitation assay (RIPA) lysis buffer containing 20 mM Tris-HCl (pH 8.0), 137 mM NaCl, 1% Nonidet-P40, 2 mM EDTA, 10% glycerol (pH 8.0), 10 μL/mL protease inhibitor cocktail, and 10 μL/mL phenylmethylsulfonyl fluoride, incubated for 5 min and centrifuged at 5,000 g for 10 min at 4°C. The supernatant was separated and its protein concentration was calculated using the DC protein assay kit (Bio-Rad, Richmond, CA) based on the Lowry method, with BSA (2 mg/mL) as the protein standard. For each sample, 30 μg protein was electrophoresed on 10% SDS-PAGE gel, transferred to a polyvinylidene-difluoride (PVDF) membrane and analyzed using the Trans-Blot SD semi-dry electrophoretic transfer cell (Bio-Rad). Membranes were blocked with 5% skimmed milk powder in PBS-Tween (0.1% PBS-T) for 1 h. The membrane was cut into two pieces, and then probed overnight at 4°C separately with anti-ANO1 (1:500, ab53212; Abcam, Cambridge, UK) or anti-β-actin (1:10,000, GTX110564; GeneTex, Iryine, CA, USA) antibodies. Excess primary antibody was removed by washing with PBS-T, and membrane was incubated with horse-radish peroxidase (HRP)-conjugated polyclonal goat anti-rabbit immunoglobulins (1:1,000, P0448; Dako, California, US) for 1 h at room temperature. All antibodies were diluted in the skimmed milk buffer. Protein bands were visualized with the ECL chemiluminescence WB Detection Reagents (GE Health Care, Little Chalfont, UK), and documented using the Image Quant LAS-4000 (GE Health Care). Quantification of bands was performed by using Image Quant TL 7.0 software (GE Health Care).

### Statistical analysis

Student's *t*-test was performed to examine the differences between two groups. All results were represented as mean ± SD, and differences were considered to be significant at *p* < 0.05. Circadian rhythms during 24- and 48-h periods were statistically analyzed by one-way analysis of variance (ANOVA), and differences were considered significant at *p* < 0.05. In addition, rhythmicity was determined by CircWave version 1.4 (Oster et al., 2006) (*p* < 0.05), and the significance (*p* < 0.05) of rhythmicity was evaluated at a 95% confidence level (α = 0.05).

## Results

### Circadian rhythmic expression of clock genes, Aqp5, and Ano1 in SGs

**Figure 1:**
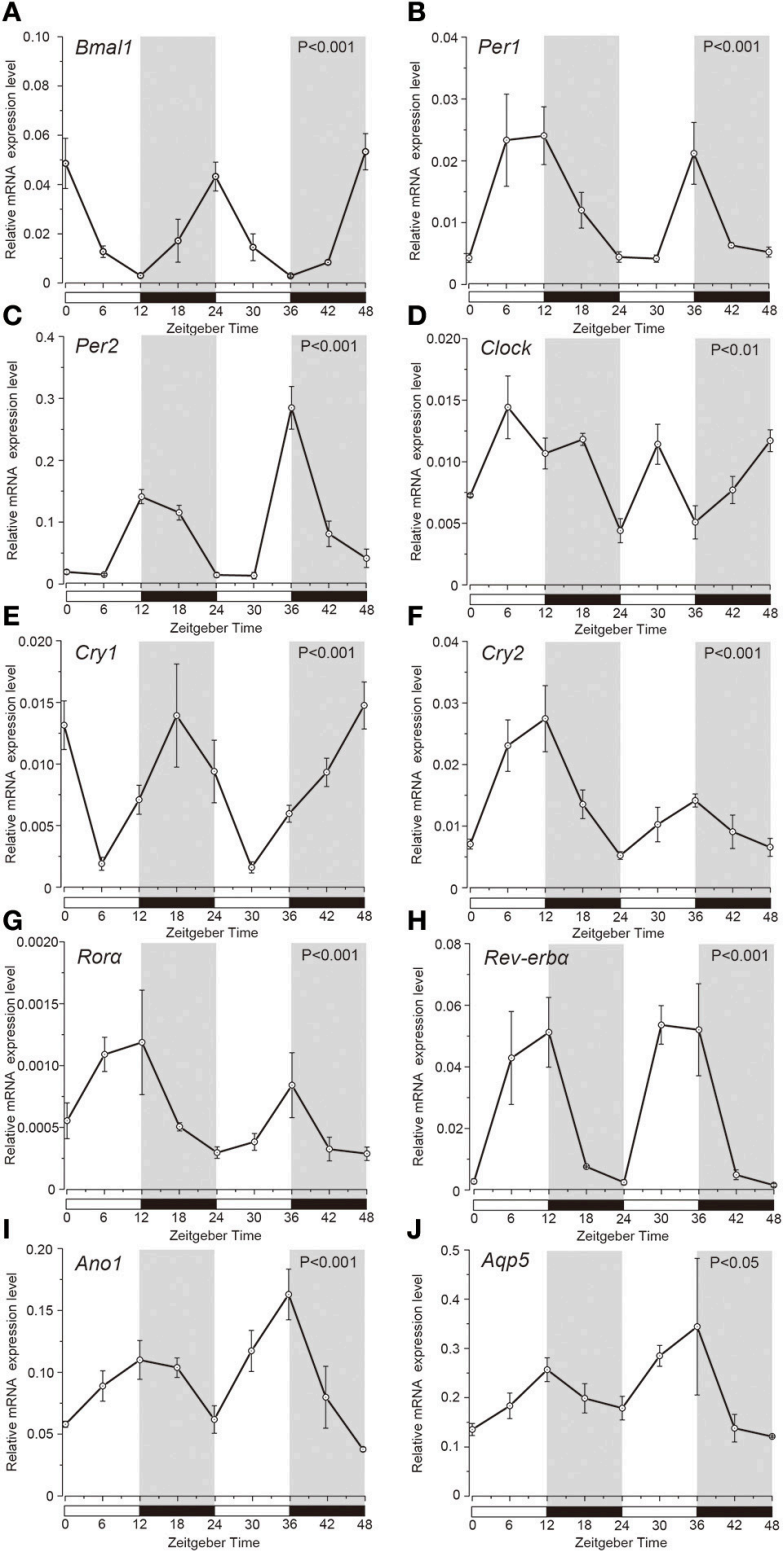
**Temporal relative mRNA expression profiles of clock-gene in whole SGs**. At the indicated Zeitgeber time (ZT: lights on at ZT0, ZT24; lights off at ZT12, ZT36), SGs were extracted from Wistar rats housed under a 12:12-h light/dark cycle for 14 days). Figure showing gene expression of **(A)** *Bmal1*, **(B)** *Per1*, **(C)** *Per2*, **(D)** *Clock*, **(E)** *Cry1*, **(F)** *Cry2*, **(G)** *Ror*α, **(H)** *Rev-erb*α, **(I)** *Ano1*, and **(J)** *Aqp5*, at 6 h intervals in ZT. The horizontal white and black bars indicate light and dark phases (shown by gray), respectively. The mRNA levels were normalized to the expression of β*-actin* mRNA and represented as the means ± SDs of three replicates per time-point (*n* = 3). *P*-values were calculated by one-way ANOVA and significance at *p* < 0.05. Rhythmicity was determined using CircWave (*p* < 0.05) at a 95% confidence level (α = 0.05). Clock genes **(A–H)** and *Ano1* **(I)** and *Aqp5* **(J)** showed rhythmic mRNA expression patterns.

Temporal relative expression profiles of the mRNAs of the clock genes, *Ano1* and *Aqp5* in the SGs were examined every 6 h from ZT0 to ZT48 (three experiments, with 27 rats in total; Figure 1). Relative expression level of *Bmal1* mRNA was significantly higher at ZT0, ZT24, and ZT48, whereas it was lower at ZT12 and ZT36 (Figure 1A). *Per1* mRNA was significantly upregulated at ZT12 and ZT36 (Figure 1B). *Per2* mRNA showed significantly higher expression at ZT12 and ZT36, and lower expression at ZT6 and ZT30 (Figure 1C). *Bmal1* expression was in antiphase with the temporal expression pattern of *Per2* with 12 h phase difference. *Clock* mRNA also showed significant rhythmic expression (one-way ANOVA, *p* < 0.01), however a clear phase variation in its expression peaks could not be observed (Figure 1D). The phase of expression of *Cry1* mRNAs also deviated by 6–12 h from the phase of *Per2* expression peaks (Figure 1E). *Cry2* mRNA showed significant upregulation at ZT12 and ZT36 and lower expression at ZT0, ZT24, and ZT48 (Figure 1F). Relative expression level of *Ror*α mRNA was significantly higher at ZT12 and ZT36 but lower, at ZT0, ZT24, and ZT48 (Figure 1G). *Rev-erb*α mRNA was significantly upregulated at ZT12 and ZT30, but showed lower expression at ZT0, ZT24, and ZT48 (Figure 1H). In addition, temporal mRNA expression profiles of water and ion secretion related plasma membrane proteins in the saliva, *Ano1*, and *Aqp5*, showed rhythmicity, with significantly higher expression observed at ZT12 and ZT36, and lower expression at ZT0, ZT24, and ZT48 (Figures 1I,J; *p* < 0.05). The expression levels of *Bmal1, Per1, Per2, Clock, Cry1, Cry2, Ror*α*, Rev-erb*α, *Ano1*, and *Aqp5* were considered rhythmic by CircWave (Figure 1).

### ANO1 expression shows a circadian rhythm

**Figure 2:**
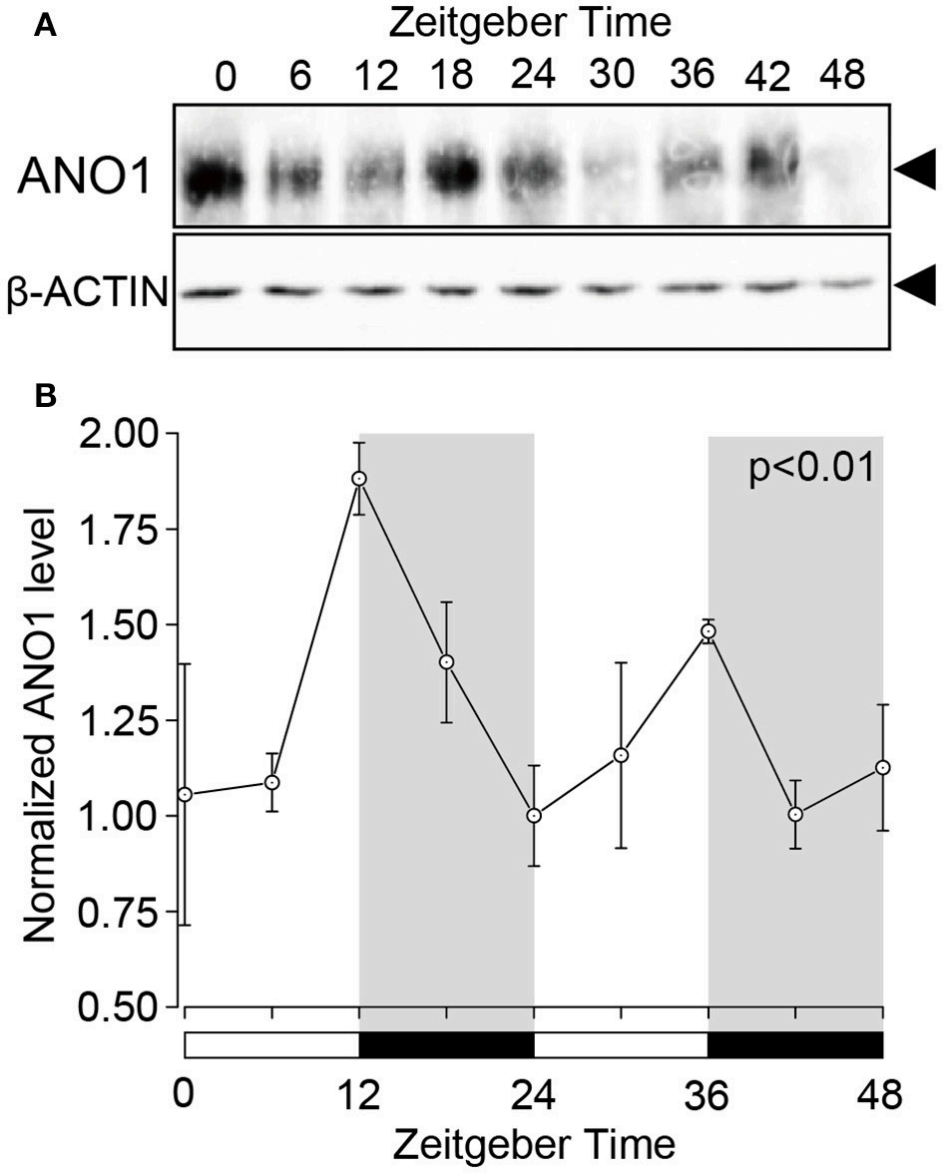
**Circadian expression of ANO1 protein in rat SGs**. **(A)** SGs were extracts at ZT0, ZT6, ZT12, ZT18, ZT24, ZT30, ZT36, ZT42, and ZT48 from three rats per time-point and subjected to western blot analysis. Total protein was separated on SDS-PAGE and probed with rabbit polyclonal ANO1 antibody (1:500). The relative density of ANO1 from the western blot was normalized against β-actin and plotted (1, density at ZT24). **(B)** Graph showing the temporal expression profile of ANO1 protein. Each point represents the mean ± SD from three independent experiments (*n* = 3). The horizontal white and black bars represent the light and dark phase (shown in gray) respectively. *P*-values were calculated by one-way ANOVA and significant differences observed at *p* < 0.01. Rhythmicity was determined using CircWave (*p* < 0.05) at a 95% confidence level (α = 0.05). The circadian expression of ANO1 showed significant oscillation patterns peaking at ZT12 and ZT36 **(B)**.

Western blot analysis revealed the circadian oscillation of ANO1 expression in the rat SGs. Temporal ANO1 protein expression was examined every 6 h from ZT0 to ZT48 (three experiments with 27 rats). A single band (~110–120 kDa) was detected for ANO1 on the SDS-PAGE gel (Figure 2A). ANO1 expression was normalized with ß-actin, a constitutively expressed internal control, every 6 h for a 48 h period. The circadian expression of ANO1 showed significant oscillation patterns peaking at ZT12 and ZT36 (ANOVA, *p* < 0.01) (Figure 2B). ANO1 expression was considered rhythmic by CircWave (*p* < 0.05, α = 0.05).

### Temporal expression pattern of clock genes in acinar and ductal cells

**Figure 3:**
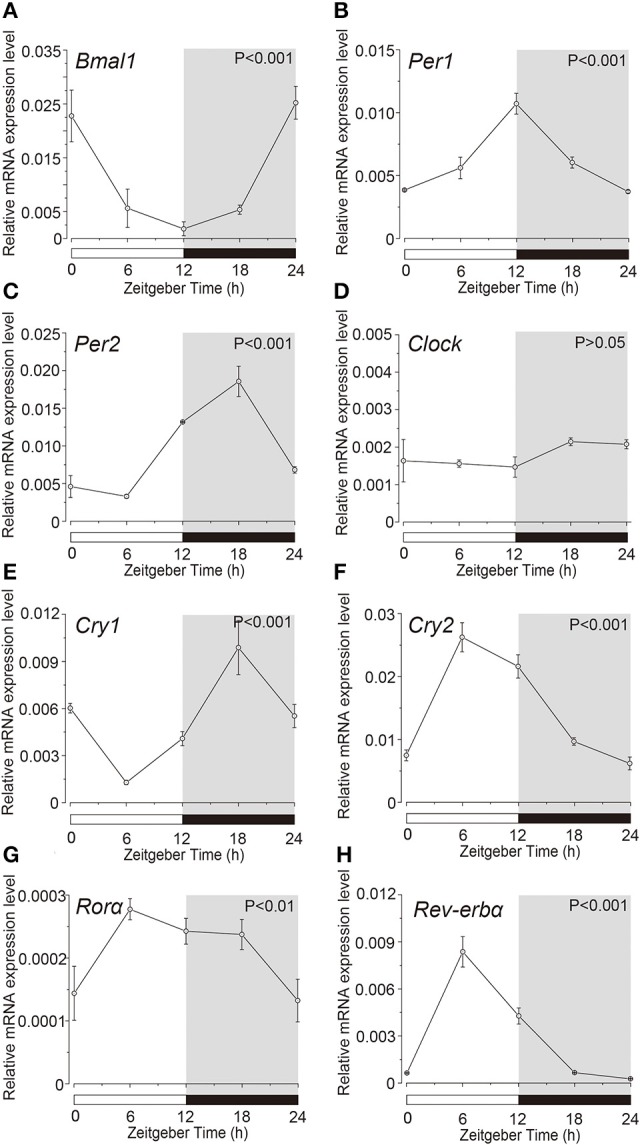
**Circadian mRNA expression profiles of clock genes in acinar cells**. Figure showing circadian rhythm in clock gene expression pattern in acinar cells by sqPCR. Relative mRNA expressions of **(A)** *Bmal1*, **(B)** *Per1*, **(C)** *Per2*, **(D)** *Clock*, **(E)** *Cry1*, **(F)** *Cry2*, **(G)** *Ror*α, and **(H)** *Rev-erb*α during 24 h period, at 6 h intervals in ZT, in the acinar cells. The horizontal white and black bars indicate light and dark phase (shown by gray). The mRNA levels were normalized to the expression of β*-actin* mRNA and expressed as means ± SD of three independent experiments per time-point (*n* = 3). *P*-values were calculated by one-way ANOVA and statistical differences at *p* < 0.05. Rhythmicity was also determined using CircWave (*p* < 0.05) at a 95% confidence level (α = 0.05). We observed rhythmic mRNA expression patterns for clock genes with the exception of *Clock* mRNA in acinar cells. Note that we used the time separating SG as a time (ZT) to evaluate temporal expression of clock gene mRNA.

**Figure 4:**
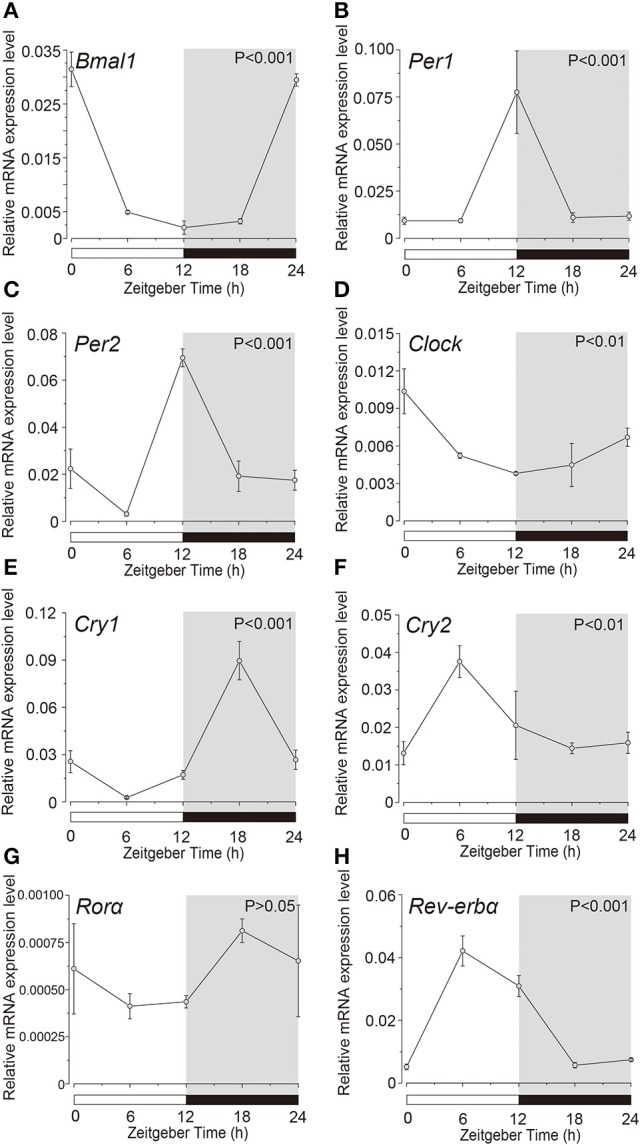
**Circadian mRNA expression profiles of clock genes in ductal cells**. Figure shows the circadian rhythm of clock gene expression pattern in acinar cells by sqPCR. Relative mRNA expression of **(A)** *Bmal1*, **(B)** *Per1*, **(C)** *Per2*, **(D)** *Clock*, **(E)** *Cry1*, **(F)** *Cry2*, **(G)** *Ror*α, and **(H)** *Rev-erb*α during a 24-h time period at 6-h intervals according to ZT in ductal cells. The horizontal white and black bars indicate light and dark phases (shown as gray), respectively. The mRNA levels were normalized to the expression of β*-actin* mRNA and expressed as means ± SDs of three independent experiments per time-point (*n* = 3). *P*-values were calculated by one-way ANOVA and results with at *p* < 0.05 were considered significant. Rhythmicity was also determined using CircWave (*p* < 0.05) at a 95% confidence level (α = 0.05). Temporal expression profiles of clock genes in the ductal cells showed circadian rhythms, except for *Ror*α. Note that we used the time separating SG as a time (ZT) to evaluate temporal expression of clock gene mRNA.

Expression profile of the clock genes in the isolated acinar and ductal cells was examined by sqPCR from three experiments with a total of 15 rats. We observed rhythmic mRNA expression patterns for *Bmal1* (Figures 3A, 4A), *Per1* (Figures 3B, 4B), *Per2* (Figures 3C, 4C), *Cry1* (Figures 3E, 4E), *Cry2* (Figures 3F, 4F), and *Rev-erb*α (Figures 3H, 4H) in both the acinar and ductal cells. Rhythmic expression was not observed in *Clock* mRNA pattern in the acinar cells (Figure 3D). However, the same was observed in the ductal cells, with ZT0 and ZT24 showing the highest, and ZT12 showing the lowest expression (Figure 4D). *Ror*α mRNA expression in ductal cells (Figure 4G) also showed no rhythmic expression, and similar results were observed in acinar cells (Figure 3G). The phases showing peak expression of *Bmal1* (Figures 3A, 4A), *Per1* (Figures 3B, 4B), *Cry1* (Figures 3E, 4E), *Cry2* (Figures 3F, 4F), and *Rev-erb*α (Figures 3H, 4H) mRNAs were almost identical in the acinar and ductal cells, however, *Per2, Clock*, and *Rev-erb*α showed different expression patterns in these cells (Figures 3C,D,G, 4C,D,G). *Per2* mRNA displayed highest expression in acinar and ductal cells at ZT18 and ZT12, respectively (Figures 3C, 4C). *Ror*α mRNA expression showed significantly high expression at ZT6 in acinar cells, whereas rhythmic expression was not observed in ductal cells (Figures 3G, 4G).

### Comparison of peak clock gene expression between acinar and ductal cells

**Figure 5:**
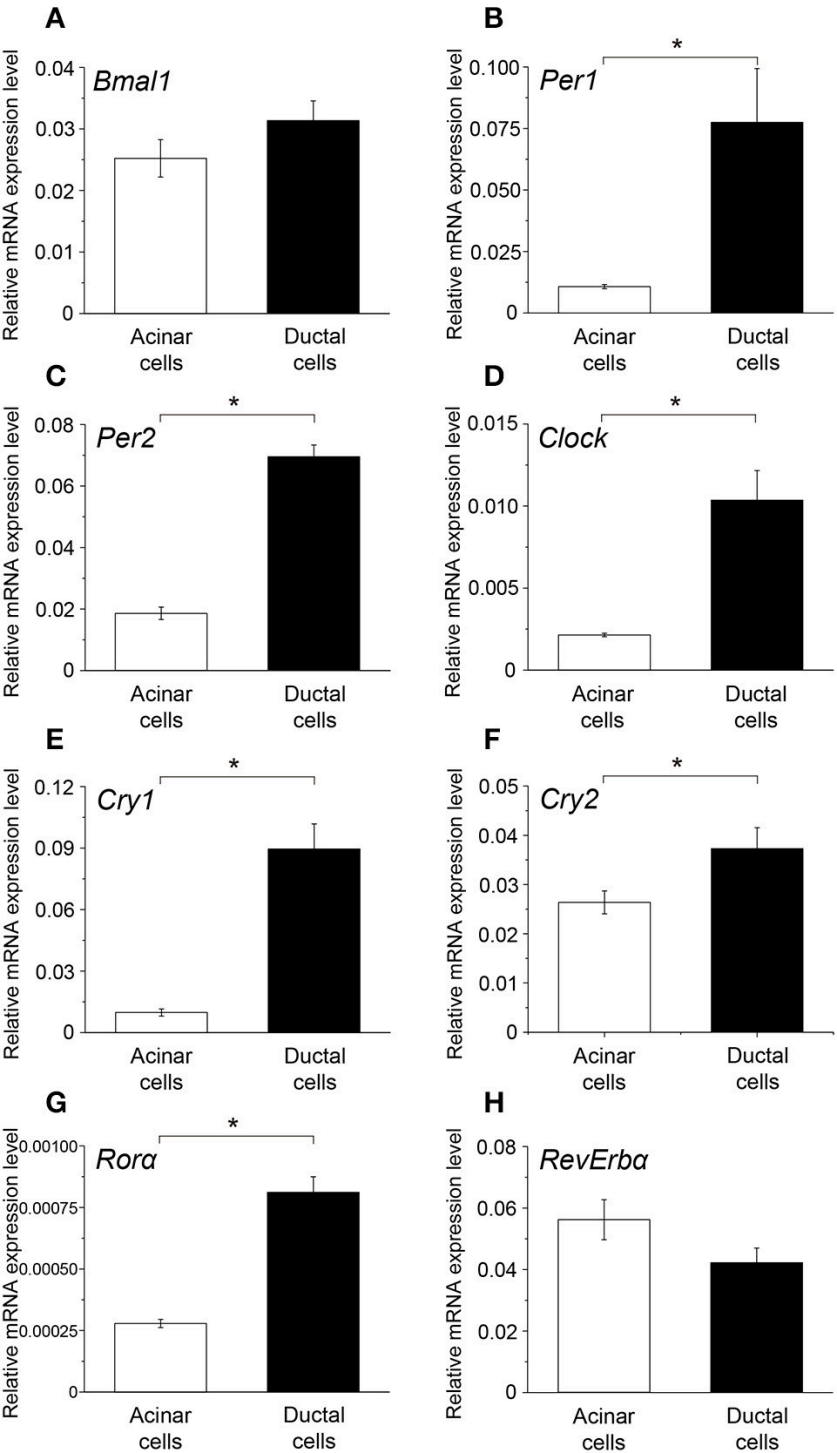
**Comparison of the peak mRNA expression of ***clock genes*** between acinar and ductal cells**. Bar graph showing the peak mRNA expression level of **(A)** *Bmal1*, **(B)** *Per1*, **(C)** *Per2*, **(D)** *Clock*, **(E)** *Cry1*, **(F)** *Cry2*, **(G)** *Ror*α, and **(H)** *Rev-erb*α in the acinar (white column) and ductal cells (black column). The mRNA levels were expressed as means ± SDs of three independent experiments (*n* = 3). Statistical analyses was performed by Student's *t*-test and statistical differences at *p* < 0.05 are indicated as “^*^” **(B–G)**.

The peak expression of *Bmal1* in ductal cells was higher than that in the acinar cells; however, the difference was not significant (Figure 5A). Additionally, the mRNA expression of *Per1, Per2, Clock, Cly1, Cry2*, and *Ror*α in ductal cells was significantly higher than that in the acinar cells (Figures 5B–G). The peak expression of *Rev-erb*α in acinar cells was slightly higher than that in ductal cells; however, this difference was not significant (Figure 5H).

## Discussion

We proved that *Bmal1, Per1, Per2, Clock, Cry1, Cry2, Ror*α, and *Rev-erb*α show rhythmic circadian expressions in SGs (Figure 1). As previously shown, the phases and peaks of *Bmal1, Per1, and Per2* expression exhibited opposite rhythms, which were shifted 12 h and repeated every 24 h. The *Cry1* expression peaks were shifted compared to those of *Per1, Per2*, and *Bmal1*. *Ror*α and *Rev-erb*α showed rhythmic circadian expression similar to that of *Per2*. The phases and peaks in their expression profiles were similar to those in the SCN and other organs (Reppert and Weaver, 2002; Ueda et al., 2005). Our results were consistent with previous studies in SGs (Furukawa et al., 2005; Zheng et al., 2012). These results suggest that rat SGs have peripheral clocks and molecular clock mechanism with negative feedback loops. Although, *Clock* expression in whole SGs showed circadian oscillations, the peaks could not be distinguished clearly. The indistinct expression pattern may be caused by the phase differences in the *Clock* expression in each acinar and ductal cell individually (see below). It has been reported that temporal expression profile of *Clock* gene in the SCN did not show any circadian rhythm, however the peripheral clocks in organs such as liver and kidney, showed circadian rhythm in *Clock* expression (Ueda et al., 2005). This is in consensus with our results.

The expression of *Ano1* and *Aqp5* mRNA in whole SGs showed circadian rhythm, which was synchronous with *Per2* expression (Figure 1). In recent studies, ANO1, CaCC, was found to express highly in the luminal membrane of the acinar cells in the SGs (Caputo et al., 2008; Yang et al., 2008). CaCC controls the apical efflux of Cl^−^ ions, which drives the transport of electrolytes and water secretion in the SGs. Transfection with mouse *Ano1* siRNA significantly reduced the peak salivary flow rate induced by muscarinic-cholinergic stimulation (Yang et al., 2008). The Activation of CaCC caused an increase in intracellular Ca^2+^ concentration, which also showed circadian rhythm (Ikeda et al., 2003). In addition, *Aqp5* knock-out mice showed decrease in expression of the tight junction proteins and water permeability, and a significant increase in the volume of the acinar cells compared to the wild type (Krane et al., 2001; Kawedia et al., 2007). In the *Aqp5* knock-out mice, saliva production by muscarinic-cholinergic stimulation was reduced by more than 60% (Yang et al., 2008). In the present study, *Aqp5* mRNA expression showed similar temporal patterns and peak times as that of *Ano1*. These results suggest, that the circadian rhythm in water secretion in SGs may be associated with water permeability, which is controlled by the circadian oscillation of *Ano1* and *Aqp5* expression. Rats displayed an increased intake of water and food during the nocturnal period (ZT12–ZT24). The expression profile of *Ano1* and *Aqp5* also correlated with their feeding and drinking behavior (Boulos and Terman, 1980; Mistlberger, 2009).

We showed that ANO1 protein expression displayed rhythmic circadian oscillations. There was no time-lag between the peak time of protein and mRNA expression (Figure 2). BMAL1 protein was transcribed from *Bmal1* mRNA without any significant time-lag (Tamaru et al., 2000). PER2 protein expression rhythm showed 4 to 6-h phase delay compared to the mRNA expression rhythm (Field et al., 2000). The result indicating that *Ano1* mRNA translated without any delay owing to factors such as phosphorylation-dependent proteolysis or shuttling by nuclear export signals.

In this study, we successfully isolated almost pure populations of acinar and ductal cells from SGs to analyze clock genes mRNA expression in each individual cell. Kallikrein, a serine protease, has been found to be localized in striated duct cells, but not in acinar cells in rat SGs (Simson et al., 1979). Immunoreactive *Egf*, *Egfr, Hgf*, *Fgf2, Tgf-*α*, Tgf-*β*1*, and *Igf1* are localized in ductal cells (Simson et al., 1979; Motoko et al., 1992; Amano and Iseki, 1993, 2001). In contrast, *Ano1* is present in acinar cells, but not in ductal cells in SGs (Yang et al., 2008). In the present study, the isolated duct-like cells showed higher kallikrein activity (Supplementary Figure 1) and *Egf*, *Egfr*, *Hgf*, *Fgf2*, *Tgf-α Tgf-β1*, and *Igf1* expression compared with *Ano1*-positive acinar-like cells (Supplementary Figure 2), indicating that we could isolate almost pure populations of acinar and ductal cells. In addition, we could not find any significant differences in *Aqp5* expression between acinar and ductal-like cells (data not shown). These results were consistent with the finding that *Aqp5* was expressed in both the apical plasma membrane in acinar cells and cells in the striated duct (Matsuzaki et al., 2012).

Acinar cells showed rhythmic expression of *Bmal1, Per1, Per2, Cry1, Cry2, Ror*α*, and Rev-erb*α, but not *Clock* (Figure 3). In ductal cells, mRNA expression of *Bmal1, Per1, Per2, Clock, Cry1, Cry2, and Rev-erb*α displayed a circadian rhythm, whereas *Ror*α did not (Figure 4). The temporal expression pattern of *Bmal1, Per1, Cry1, Cry2*, and *Rev-erb*α in both the acinar and ductal cells, isolated from the SGs, had very similar circadian rhythms. The rhythmic expression pattern of *Clock* was indistinct in whole SGs and acinar cells. However, ductal cells showed rhythmic *Clock* mRNA expression, similar in pattern to *Bmal1*. The obscurity in *Clock* rhythmic expression in whole SGs might be due to overlapping cycles of mRNA expression in individual acinar and ductal cells. Phase difference in mRNA expression for *Per2* and *Ror*α was also observed between acinar and ductal cells. The peak mRNA expression time for *Per2* in whole SGs, corresponded with that in the isolated ductal cells. The peak time of expression for *Ror*α in whole SGs did not coincide with that in acinar and ductal cells. Variable oscillatory expressions of *Per1*, a paralog of *Per2*, has been observed in individual cell in the SCN (Yamaguchi, 2003). These results suggest that temporal expression patterns of clock gene mRNAs in whole SGs are established by the combination of their expression profiles in both the acinar and ductal cells. *Per1, Per2, Clock, Cry1, Cry2*, and *Ror*α showed high expression in ductal cells (Figure 5). The mRNA expression levels of *Bmal1* and *Rev-erb*α were not significantly different. These results are also consistent with previous studies demonstrating the localization of *Per2* mRNA expression in mice SGs by *in situ* hybridization (Zheng et al., 2012). Our results also confirmed the localization of mRNAs encoding clock genes in acinar and ductal cells. In addition, we revealed that there were broad differences in clock gene expression levels between acinar and duct cells.

To confirm the relationship between clock genes and *Ano1* and *Aqp5* expression, we studied their DNA sequences. The promoters of rat *Ano1* and *Aqp5* have an enhancer box (E-box) binding sequence. The E-box is a DNA response element that acts as a protein-binding site and is necessary for the binding of BMAL1-CLOCK heterodimer to the promoter region. E-box plays a major role in regulating the transcription of *Per2* and *Cry1* (Bozek et al., 2009). A recent study has identified the E-box protein-binding sequence on the *Aqp5* promoter in humans and mice (Zheng et al., 2012). In rats, the E-box element has been identified from −160 to +69 on the *Aqp5* promoter (Nomura et al., 2007). The existence of E-box in the promoter region is a characteristic of clock-controlled genes (CCGs), which show rhythmic expression and contain clock gene binding motifs, in the promoter (Bozek et al., 2009). Thus, *Ano1* and *Aqp5* may be putative CCGs and key targets of the BMAL1-CLOCK heterodimer in SGs.

In conclusion, we showed circadian rhythmic expression of *Bmal1, Per1, Per2, Clock, Cry1, Cry2, Ror*α, and *Rev-erb*α mRNAs in not only whole SGs, but also isolated acinar and ductal cells. From our personal communications, *Bmal1* mRNA also showed rhythmic expression in a salivary ductal cell line (He et al., 1998). Although we could not exclude the possibility of contributions of a master clock in the suprachiasmatic nucleus to circadian rhythmic salivation, cells in the salivary gland may have independent peripheral clocks. Further studies are needed to clarify the involvement of the suprachiasmatic nucleus in the salivation rhythm by using *in vivo* experiments, such as monitoring of salivary secretion following section of nervous input to the SGs. Clock genes may also regulate the circadian rhythmic oscillatory expression of *Ano1*, and *Aqp5* mRNA and ANO1 protein, and may play an important role in the physiological nocturnal salivary secretion including water and electrolyte secretion. In addition, predominant rhythmic expression of clock genes in ductal cells may play an important role in modifying the ionic composition of the saliva, by reabsorbing and/or secreting ions during salivation.

## Author contributions

RS, YS, and NS conceived and designed the experiments. RS performed all the experiments. RS and YS conceived the ideas, and wrote the paper. RS, YS, MS, MK, MT, YI, and NS contributed to the analysis of results and preparation of the figures and tables. All authors read and approved the results and the final manuscript.

### Conflict of interest statement

The authors declare that the research was conducted in the absence of any commercial or financial relationships that could be construed as a potential conflict of interest.
