# Supplementary material for: Temporal Expression Patterns of Clock Genes and Aquaporin 5/Anoctamin 1 in Rat Submandibular Gland Cells
Source: Front Physiol. 2017 May 23;8:320. doi: 10.3389/fphys.2017.00320 (PMC5440558; doi:10.3389/fphys.2017.00320)
Supplement: Supplementary file 1 [file Table1.DOCX]

Supplementary Table 1. Primer sequences for sqPCR

Gene Name 5’-sequence-3’ GenBank Number

*β-actin* Forward GGAGATTACTGCCCTGGCTCCTA NM_031144.3

Reverse GACTCATCGTACTCCTGCTTGCTG

*Bmal1* Forward TTCATGAACCCGTGGACCAA NM_024362.2

Reverse CCCTGGAATGCCTGGAACA

*Per1* Forward GCCTCAGGCCCTCGATGTAA NM_001034125.1

Reverse CGAGTGGCCAGGATCTTGAA

*Per2* Forward TCTCAGAGTTTGTGCGATGATTTG NM_031678.1

Reverse CACTGGGTGAAGGTACGTTTGG

*Clock* Forward ACACAGCCAGCGATGTCTCAA NM_021856.1

Reverse CATGGCTCCTAACTGAGCTGAAAG

*Cry1* Forward CGGCGACCTATGGATCAGTTG NM_198750.2

Reverse TCCCAGCATTGATGCTCCAG

*Cry2* Forward GTTCTTCCACTGCTACTGCCCTG NM_133405.2

Reverse CTTAGCGGCCTTCTGAACCGAC

*Rorα* Forward CTCCAGCCGAGGTGACTTTATCATC XM_008766407.1

Reverse ACTTTAGCTCGTGGGTTCTCCTTC

*Rev-erbα* Forward GACATGACGACCCTAGACTCCAAC NM_001113422.1

Reverse AGTCAGGGACTGGAAGCTGC

*Ano1* Forward TCAAAGGCCGGTTTGTTGGTCG NM_001107564.1

Reverse GGCGAAGGGTTCGAGGTTGAAG

*Aqp5* Forward GCCGTCAATGCGCTGAACAAC NM_012779.1

Reverse CATGGAACAGCCGGTGAAGTAGATC

*Bmal1*, aryl hydrocarbon receptor nuclear translocator-like protein 1; *Per1*, period 1; *Per2*, period 2; *Clock*, circadian locomotor output cycles kaput; *Cry1*, cryptochrome circadian clock 1; *Cry2*, cryptochrome circadian clock 2; *Rorα*, RAR-related orphan receptor alpha; *Rev-erbα*, nuclear receptor subfamily 1, group D, member 1; *Ano1*, Anoctamin 1; *Aqp5*, Aquaporin 5; GenBank Number; the Accession number of NIH genetic sequence database.
