# Supplementary material for: Temporal Expression Patterns of Clock Genes and Aquaporin 5/Anoctamin 1 in Rat Submandibular Gland Cells
Source: Front Physiol. 2017 May 23;8:320. doi: 10.3389/fphys.2017.00320 (PMC5440558; doi:10.3389/fphys.2017.00320)
Supplement: Supplementary file 2 [file Table2.DOCX]

Supplementary Table 2. Primer sequences for sqPCR in isolation of acinar and ductal cells experiment

Gene Name 5’-sequence-3’ GenBank Number

*Egf* Forward TGCCTTGCCCTGACTCTAC NM_012842.1

Reverse AGCCAATGACACAGTTGCAC

*Egfr* Forward GTGTCAAGACCTGCCCTTCG NM_031507.1

Reverse GGGATCTTTGGCCCTTCTGG

*Hgf* Forward TGCAACGGTGAAAGCTACAGAG NM_017017.2

Reverse ATTTGTGCCGGTGTGGTGT

*Fgf2* Forward ACGGCGTCCGGGAGAA NM_019305.2

Reverse ACACTCCCTTGATGGACACAACT

*Tgf-α* Forward ACCACGCTCTTCTGTCTACTG NM_012671.2

Reverse CTTGGTGGTTTGCTACGAC

*Tgf-β1*Forward GACGTCACTGGAGTTGTCCG NM_021578.2

Reverse ACTGATCCCATTGATTTCCACG

*Igf1* Forward GCCGTCAATGCGCTGAACAAC NM_001082477

Reverse CATGGAACAGCCGGTGAAGTAGATC

*Egf*, epidermal growth factor; *Egfr*, epidermal growth factor receptor; *Hgf*, hepatocyte growth factor; *Fgf2*, fibroblast growth factors 2; *Tgf-α*, transforming growth factor α; *Tgf-β1*, transforming growth factor-β type I; *Igf1*, insulin-like growth factor 1; GenBank Number; the Accession number of NIH genetic sequence database.
