# Supplementary material for: Temporal Expression Patterns of Clock Genes and Aquaporin 5/Anoctamin 1 in Rat Submandibular Gland Cells
Source: Front Physiol. 2017 May 23;8:320. doi: 10.3389/fphys.2017.00320 (PMC5440558; doi:10.3389/fphys.2017.00320)
Supplement: Supplementary file 3 [file Table3.DOCX]

**Temporal Expression Pattern of Clock Genes and Aquaporin 5　/Anoctamin 1 in Rat Submandibular Acinar and Ductal Cells**

**Ryouichi Satou^1^, Masaki Sato^2^, Maki Kimura^2^, Yoichi Ishizuka^1^, Masakazu Tazaki^2^,**

**Naoki Sugihara^1^, and Yoshiyuki Shibukawa^2＊^**

^1^Department of Epidemiology and Public Health, Tokyo Dental College, Tokyo, Japan

^2^Department of Physiology, Tokyo Dental College, Tokyo, Japan

* Correspondence:

Yoshiyuki Shibukawa

Department of Physiology, Tokyo Dental College

2-9-18, Misaki-cho, Chiyoda-ku, Tokyo, Japan 101-0061,

Teleppone: +81-03-6380-9567, Fax: +81-03-6380-9346

E-mail: yshibuka@tdc.ac.jp

**Running title: Clock genes in submandibular glands**

**Keywords: Aquaporin, circadian rhythm, clock gene, secretion, chloride channel, transport**

**Article types: Original Research Article**

**APPENDIX: MATERIAL AND METHODS**

***Isolation of rat SG acinar and ductal cells***

Submandibular acinar and ductal cells were isolated following the protocol by Sakai et al and Nezu et al., with modifications (Sakai et al., 2002; Nezu et al., 2000). Briefly, rats were anesthetized with pentobarbital sodium, containing isoflurane (3%). SGs were dissociated from anesthetized rat at each time point (ZT0 to ZT48 in maximum). SGs were finely minced and washed in Krebs-Ringer bicarbonate (KRB) solution containing 115 mM NaCl, 5.4 mM KCl, 1.35 mM CaCl_2_, 0.6 mM MgSO_4_, 25 mM NaHCO_3_, 0.96 mM NaH_2_PO_4_, 11 mM glucose, 5 mM HEPES (pH 7.4, adjusted with NaOH). The minced tissue was incubated for 40 min with constant shaking (30 cycles/min) at 37 ℃ in 5 mL KRB solution containing collagenase type II (0.2 mg/mL; Worthington, Lakewood, USA), hyaluronidase (0.2 mg/mL; Sigma, St. Louis, USA) and bovine serum albumin (BSA, 0.5%), followed by trituration. After dispersion, the cell suspension was passed through a 150 μm nylon mesh and centrifuged at 100 g for 5 min. The pellet was resuspended in 2.5 mL Ca^2+^/Mg^2+^-free KRB solution containing 0.1% BSA. 5 mL of 40% isotonic Percoll (GE Healthcare, Little Chalfont, UK) was prepared and 2.5 mL cell suspension was layered on top of it, followed by centrifugation at 4000 g for 10 min. After centrifugation, the cells were found to separate into two distinct populations. These cells were immediately subjected to the mRNA expression or measurement of kallikrein activity. All the cell isolation protocols were carried out within 2 h.

***Measurement of kallikrein activitiy***

Kallikrein activity was measured (Supplementary Figure 1) according to the method by Geiger et al. (Geiger et al., 1980), using *N*α-benzoyl-dl-arginine *p*-nitroanilide (BANA). Briefly, 400 μL isolated cell sample that remained on top of the Percoll gradient was mixed with 100 μL of 0.5 M tris(hydoroxymethyl)aminomethane hydrochloride (pH 8.5) in a 500 μL cuvette and pre-warmed to 37 ℃. 200 μL BANA, the chromogenic substrate for kallikrein (2 mg/mL; Wako Pure Chemicals, Osaka, Japan) was added and absorption was measured at 405 nm for 10 minutes at 30 s intervals, and plotted using SpectraMax M5e (Molecular Devices, California, USA).

**References**

Geiger, R., Fink, E., Lottspeich, F., Henschen, A., and Fritz, H. (1980). Human urinary kallikrein—Biochemistry and physiological studies. *Fresenius Z. Für Anal. Chem.* 301, 172–173. doi:10.1007/BF00467808.

Nezu, A., Tanimura, A., and Tojyo, Y. (2000). Isolation of rat submandibular ductal cells by Percoll centrifugation. *Higash Nippon Dent. J.* 19, 15–22.

Sakai, T., Michikawa, H., Furuyama, S., and Sugiya, H. (2002). Methacholine-induced cGMP production is regulated by nitric oxide generation in rabbit submandibular gland cells. *Comp. Biochem. Physiol. B Biochem. Mol. Biol.* 132, 801–809.
