# Supplementary figures and images for: Temporal Expression Patterns of Clock Genes and Aquaporin 5/Anoctamin 1 in Rat Submandibular Gland Cells
Source: Front Physiol. 2017 May 23;8:320. doi: 10.3389/fphys.2017.00320 (PMC5440558; doi:10.3389/fphys.2017.00320)

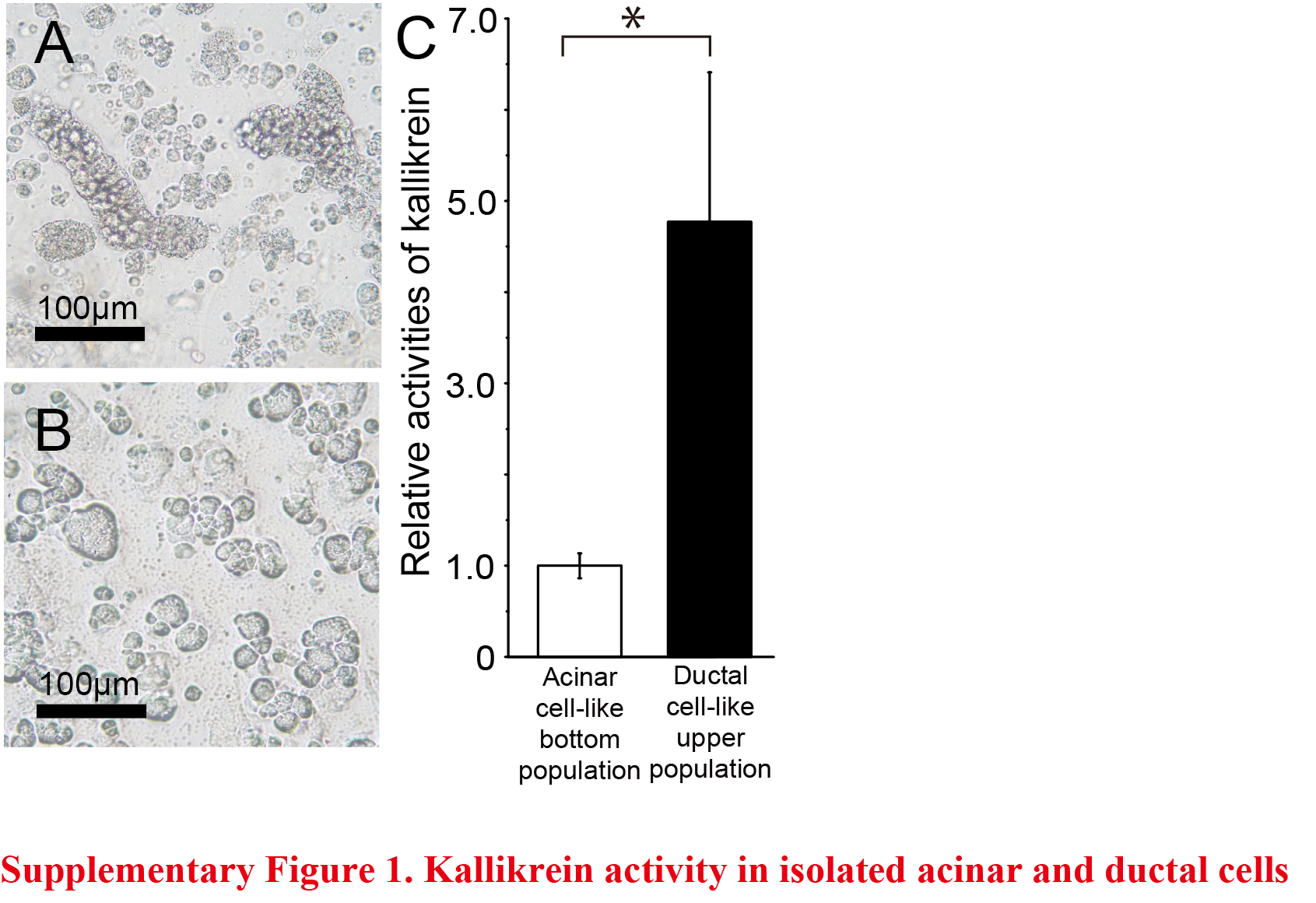

Supplement: Supplementary file 4 [file Image1.TIF]

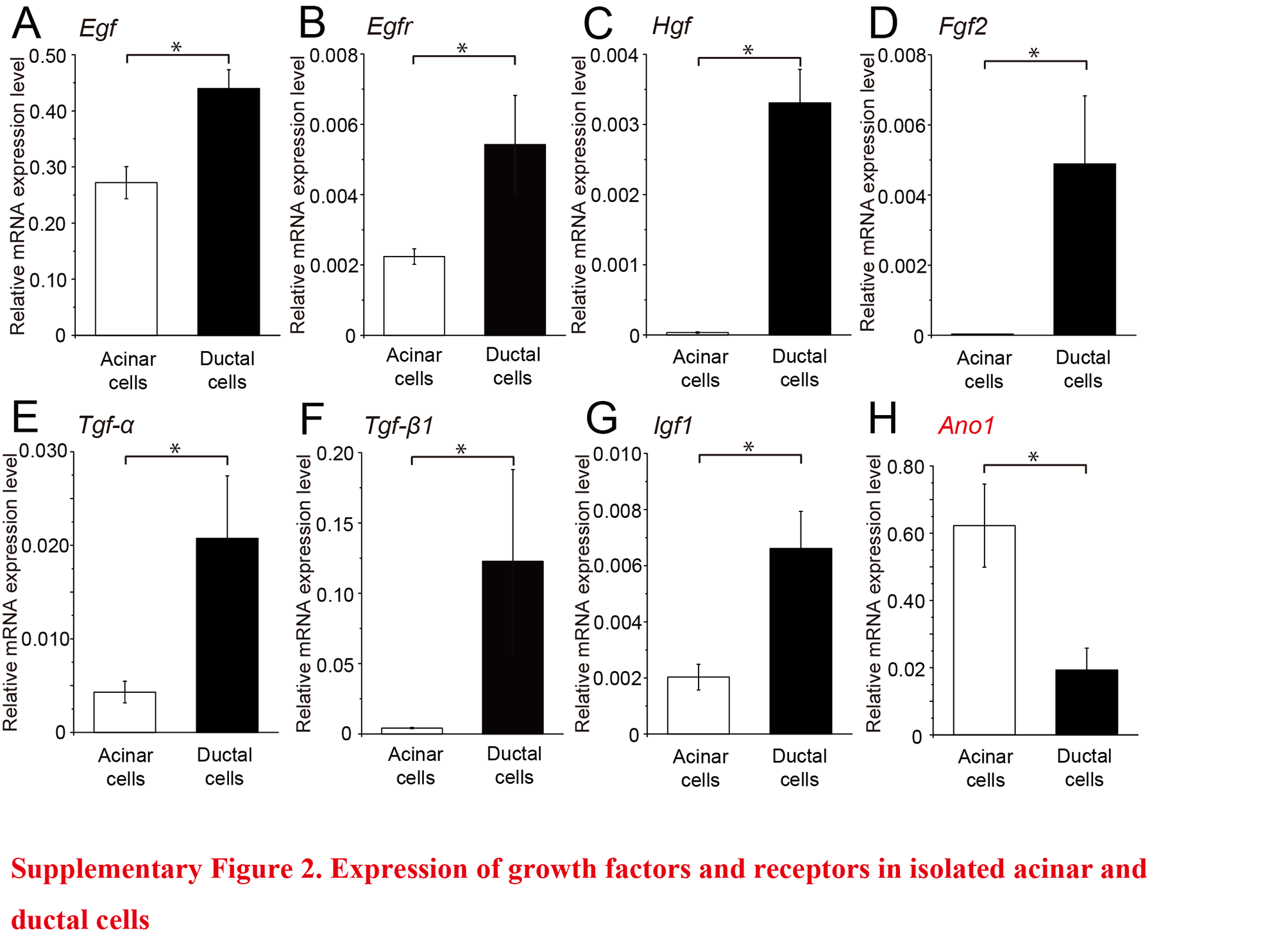

Supplement: Supplementary file 5 [file Image2.TIF]
